# Work Related Musculoskeletal Pain in Golf Caddies—Johannesburg, South Africa

**DOI:** 10.3390/ijerph17103617

**Published:** 2020-05-21

**Authors:** Jennica Garnett, Felix Made, Nonhlanhla Tlotleng, Kerry Wilson, Nisha Naicker

**Affiliations:** 1 School of Population and Public Health, University of British Columbia, Vancouver, BC V6T 1Z4, Canada; jennica.garnett@alumni.ubc.ca; 2 Epidemiology and Surveillance Section, National Institute for Occupational Health (NIOH), A Division of National Health Laboratory Service (NHLS), Johannesburg 2000, South Africa; FelixM@nioh.ac.za (F.M.); NonhlanhlaT@nioh.ac.za (N.T.); KerryW@nioh.ac.za (K.W.); 3 School of Public Health, University of the Witwatersrand, Johannesburg 2000, South Africa; 4 Department of Environmental Health, Faculty of Health Science, University of Johannesburg, Johannesburg 2000, South Africa

**Keywords:** caddies, informal job, musculoskeletal pain, South Africa, golf, occupational health

## Abstract

Golf is an important and growing industry in South Africa that currently fosters the creation of an informal job sector of which little is known about the health and safety risks. The purpose of the study is to investigate the prevalence and significance of musculoskeletal pain in male caddies compared to other golf course employees while holding contributing factors such as socioeconomic status, age, and education constant. Cross-sectional data were collected and analyzed from a convenience sample of 249 caddies and 74 non-caddies from six golf courses in Johannesburg, South Africa. Structural interviews were conducted to collect data on general demographics and musculoskeletal pain for two to three days at each golf course. On average, caddies were eight years older, had an income of 2880 rand less a month, and worked 4 h less a shift compared to non-caddies employed at the golf courses. Caddies were approximately 10% more likely to experience lower back and shoulder pain than non-caddies. Logistic regression models show a significantly increased adjusted odds ratio for musculoskeletal pain in caddies for neck (3.29, *p* = 0.015), back (2.39, *p* = 0.045), arm (2.95, *p* = 0.027), and leg (2.83, *p* = 0.019) compared to other golf course workers. The study findings indicate that caddying, as a growing informal occupation is at higher risk for musculoskeletal pain in caddies. Future policy should consider the safety of such a vulnerable population without limiting their ability to generate an income.

## 1. Introduction

Golf has shown tremendous growth worldwide, becoming one of the largest sports-related travel markets [1]. Offering 345 playable days a year and considerably low membership costs and green fees, South Africa has developed rapidly into a competitive golf industry market [2]. With one of the highest average number of full-time employees globally, at 42 employees per 18-hole course, South Africa’s golf industry has the potential for significant economic growth and opportunity. Unfortunately, South Africa’s golf courses have the lowest salary costs globally, making up only 23% of their operating budget compared to 30–40% in Europe [2]. With impending development and future business performance, it is imperative that the occupational health of the golf course workers be investigated.

Caddying is considered a low-skill job with poor working conditions. In South Africa, caddies are part of the informal workforce, as they are not legally employed by the golf course, they arrive at the course in hopes of acquiring employment by the golfer. This means that caddies could be waiting at the golf course all day, and not work a single round of golf. Informal workers generally have no control over their work environment, while the formal economy is governed by policies and legislation. Despite the caddies being informal workers, the expectations from the golf course are similar to those of regular employees. Most caddies are required to wear uniforms and abide by company codes of conduct and performance. In contrast, the golf courses are not responsible for maintaining adequate working conditions or a safe working environment. Adequate working conditions would include access to protective equipment such as shoes, hats, gloves, sunscreen, and basic human necessities such as clean drinking water or a space to break or rest [3]. A safe working environment, for example, would include policies and support to protect against verbally abusive patrons [3]. This unique circumstance makes caddies a vulnerable population susceptible to exploitation and injury.

Caddies are traditionally exposed to many risk factors associated with musculoskeletal pain and other physical problems during their work. Given the unique structure of their employment, serious lack of occupational health and safety equipment and inconsistent working hours, the prevalence of musculoskeletal pain may be vastly different than formally employed caddies. A study investigating caddies in South Korea found that 44.8% of caddies complained of musculoskeletal pain or ailments resulting from the repetitive standing, walking, and carrying golf bags as required by their job [4]. This study is comparable because caddies in South Korea have an informal employment structure and limited control over their occupational health and safety equipment and environment. To our knowledge, there have been no studies investigating musculoskeletal pain experienced by caddies in South Africa. Previous international study of musculoskeletal pain in caddies may not be comparable to the South African sample because these studies investigated caddies with formal employment and regular extended working hours. Knowledge of the rates and contributing factors related to occupational musculoskeletal pain specific to the golf industry in South Africa could provide much-needed support for policy development to increase preventative measures to this emerging profession. 

The aim of this cross-sectional study was to assess the prevalence and estimate the adjusted odds-ratio of musculoskeletal pain in male caddies compared to other golf course workers and investigate the association with sociodemographic characteristics and work activities. Exploring the relationship between occupation and pain among caddies is a useful first step toward the development of appropriate interventions and policies. 

## 2. Materials and Methods 

### 2.1. Participants

Seven golf courses were selected and approached, with six agreeing to participate in the study. Convenience sampling was used to survey caddies at each golf course located in Johannesburg, South Africa. The study was conducted over a 2- to 3-day period at each golf course to increase the probability of capturing the greatest number of individuals and decrease non-response rate. All individuals present on the day of data collection, and who consented, participated in the study. Of the 329 participants registered, 323 completed the survey, 249 of them identified as caddies, and 74 identified as non-caddies. This study included only male individuals because all caddy workers were males. Therefore, no females were included in the non-caddies group, which comprised of groundskeepers, restaurant staff, and administrative employees who were formally employed by the golf course. 

### 2.2. Measurements

Structured face-to-face interviews were performed by trained fieldworkers, with local language translation possible, using electronic RedCap data processing software after informed consent was obtained. The questionnaire consisted of 268 detailed questions about socio-demographics (including age, education, living costs, income, and food security), occupational history (length of shifts, number of shifts a week), occupational exposures (history of injuries), alcohol and drug use, baseline health, healthcare access, and mental health screening. 

Musculoskeletal pain was measured by structured questions adapted from the validated Nordic Musculoskeletal Questionnaire [5]: such as “have you at any time in the last 12 months had trouble (ache, pain, discomfort, numbness) in the neck” and “have you had trouble at any time during the last 7 days (with neck pain).” Data about pain were also collected from different areas of the body including shoulder, elbow, hand and wrist, upper back, lower back, hip, knee, and ankle. Participants were able to answer yes or no, and specify the right, left, or both appendages if applicable. Participants were also asked if they felt their pain was due to their occupation, and if so, what they felt the contribution factors or actions were. This was an open-ended question which was captured in free text by the interviewer.

### 2.3. Data Analysis

Descriptive statistics such as means and standard deviations were used to summarize continuous variables, while categorical variables were presented in frequency and percentages. Potential confounding or predictor variables were identified; age, body mass index (BMI), chronic illness, education, primary provider, number of dependents, housing, monthly income, days a week worked, and average length of shift. Other variables such as smoking status, alcohol consumption, distance walked during shift, weight of golf bag, and access to drinking water were considered but were not viable due to significant null response (>20% of participants). The variables for pain were compared between occupational groups using descriptive statistics.

All statistical analysis was conducted in R software (University of Auckland, Auckland, New Zealand). All univariate and bivariate analysis was completed prior to initiating the regression analysis and a significance level of 5% was applied to all tests. 

A logistic regression model was fitted to investigate the effect of working as a caddy on developing musculoskeletal pain. For these models, the categorical variables for pain were condensed into four categories–neck, back, arm, and leg. Shoulder, elbow, hand, and wrist pain were joined to create the variable arm pain. Upper and lower back pain were joined to create the variable back pain. Hip, knee, and ankle pain were joined to create the variable leg pain. Pain was not differentiated by how many of the limbs or areas it was present in, a single yes in any area identified was coded as positive for pain. 

Four models were created, each focusing on a separate location of pain; neck, back, arm, and leg. Initially, a simple regression model was built to determine the unadjusted work type category (caddy or non-caddy) effect on musculoskeletal pain, to which variables were added individually and analyzed. The variables were added in the same order for each model and followed the listed groupings: demographics, health indicators, and job-related factors. Variables were kept in the model if they produced a 10% or larger change in the work type coefficient or the work type standard error, and the Akaike’s Information Criteria (AIC) did not increase by more than 2. Using these parameters, “primary income” and “number of dependents” were not kept in any of the models. When “chronic illness” was added to the arm pain model, there was no change in AIC, work type coefficient, or work type standard error, however due to the conceptual link between pain, injury, and chronic disease it was kept in the model. When “BMI” was added to the leg pain model, it did not cause a 10% or larger change in B1 or B1SE, but the AIC decreased from 405 to 395, because of this it was kept in the model. “days worked a week” was only significant in the leg pain model, therefore was the only model that kept this variable. After the final models were created, variables “primary income”, “number of dependents”, and “days worked a week” were readded to the models where applicable and compared to the final model. Re-adding these variables in all 4 pain models, caused no significant changes to work type coefficient, and increased AIC. The sample size used for each model is indicated, as some cases were removed due to missing data. 

## 3. Results

### 3.1. Sample Demographics, Health Behaviours, and Job-Related Factors

A description of the sample population is presented in Table 1. Fifty percent of the caddies had a monthly income of less than 2849 rand ($182) per month. The non-caddies earned nearly double with a mean of 5729 rand ($367). This disparity in monthly income highlights the socio-economic instability of caddies and in previous reports has been linked to food insecurity [3]. Non-caddies worked five to seven days per week, with a median of five days and eight hours per day. On the contrary, caddies worked a median of three days a week for five hours per day. The informal nature of caddy’s work means that caddies often wait at the golf course for an opportunity to work, so the time reflected in a typical working day does not indicate how much time is spent at the golf course waiting for work.

**Figure 1:**
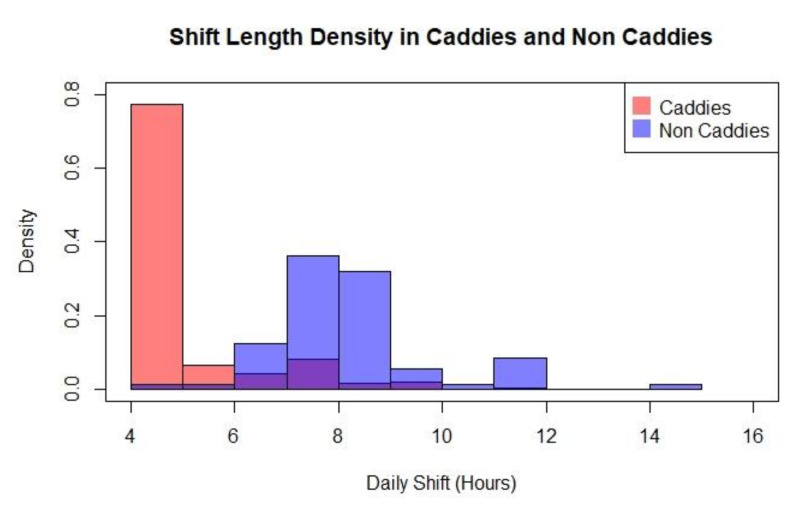
Shift Length Density in Caddies and Non-Caddies.

**Figure 2:**
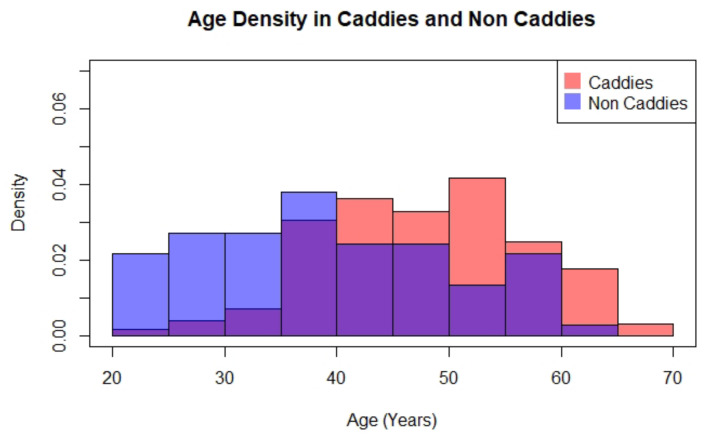
Age Density in Caddies and Non-Caddies.

The difference in regular working hours between the two occupational categories is shown in Figure 1. Overall, caddies have shorter working hours than non-caddies. The two occupational groups also differ in age, caddies have a mean age of 48 compared to non-caddies. Caddies represent a more mature population shown in Figure 2.

### 3.2. Analytic Comparisons

Overall caddies reported a higher prevalence of musculoskeletal pain, the most commonly affected areas being lower back (38%), shoulders (35%), and ankles (32%). Of the caddies that responded, 60% attributed carrying heavy golf bags as the cause of pain. Walking was identified in 33% of responding cases as their self-identified action causing pain. Of those caddies who reported lower back and ankle pain, over 40% were forced to take time off because of the discomfort. The non-caddies reported a lower prevalence of back (29%), shoulder (24%), and neck (22%) pains. Of the non-caddies that responded, 9% responded that they attributed carrying golf bags as the cause of pain, while walking was identified in 13% of respondents. Some areas for self-identified causes of pain were chemicals (4%), ergonomic (26%), and other (48%).

### 3.3. Logistic Regression Models

The logistic regression models were created to quantify the effect that being a caddy has on the odds of developing a musculoskeletal pain compared to other golf course workers. Data from the entire sample was placed in each of the models created, each estimating the odds for a different pain location, work type category was the only variable consistently significant in each model, holding all other variables constant. The work category coefficients and odds ratios are captured in Table 2. Ultimately, the odds of a caddy experiencing musculoskeletal pain were 2.39 to 3.29 times the odds of a non-caddy, depending on pain location. 

## 4. Discussion

Musculoskeletal conditions, including pain, cause a significant global burden [6]. The Global Burden of Disease 2010 study showed that lower back pain ranked highest for disability and sixth for overall burden, while neck pain ranked fourth highest for disability and 21st for burden [6]. Despite the increased focus on musculoskeletal pain globally, there remains a significant deficit in research specific to South African’s working-age population and even less investigating the specific mechanisms of pain in informal occupations. To our knowledge, there has not been a recent national South African survey that estimates the prevalence of musculoskeletal pain. The first steps in understanding the magnitude of the problem is increasing the related research especially among vulnerable populations such as low socioeconomic groups [7,8]

Caddies represent a vulnerable population of men working in an informal capacity, with little structure in income or consideration of safety. The role of the work environment in developing musculoskeletal pain in caddies has not been previously investigated in South Africa. The caddies in South Africa are not working long hours but waiting for hours and sometimes days for the opportunity to be hired by a golfer. This presents a much different environment for pain and injury than a traditional caddy role which may include multiple games per day. In contrast, other studies have investigated musculoskeletal pain in caddies but in a formally employed role with substantially improved equipment and different work environments. 

Caddies are likely entering the job from a place of little employment options and limited income stability [7]. The socio-economic, health, and job factors were pivotal in determining the direction of influence in this relationship. The adjusted odds ratios present a strong case that the physical work being completed by caddies is affecting their rate of musculoskeletal pain compared to other golf course workers. The most common locations for musculoskeletal pain in caddies are the shoulder, ankle, and lower back [3]. Caddies have self-identified that these pain locations are likely related to actions they take to perform their job, which includes walking approximately six km per game and carrying a golf bag of approximately 15 kg [9]. Carrying a golf bag over ones’ shoulder puts direct pressure and strain on the shoulder and neck muscles and alters a person’s upright posture. Walking with a heavy golf bag demands greater muscle activation and overloading these muscles can lead to musculoskeletal pain [9]. Gosheger et al. found that carrying a golf bag for approximately four to five hours is physically demanding and commonly results in shoulder, back, and ankle injuries in persons who carried their bag on a regular basis [9]. 

Golf tourism has increased in South Africa and stands to continue to grow [10]. The golf courses charge relatively low green fees and membership fees and spend little on labour and wages compared to global competitors. Many golf courses do not offer motorized or pushcarts for the golf bags and so capitalize on workers presenting at the course to be hired directly by the golfer. This allows golf courses to take minimal or no responsibility for the safety of the persons that work on their course. Most courses expect caddies to wear a uniform and provide them; however, do not provide equipment for adequate occupational safety, such as shoes, hats, or gloves.

This does not go without considering the current relationship between the golf course and caddy. Caddies currently have the freedom to create their own schedules and select persons that they work for, these types of advantages may provide reasons not to push for a more structured form of employment [11]. This reasoning has been highlighted previously in a similar case in India, in which caddies did not want to seek formal employment and preferred the current informal structure with suggestions for minor changes [11]. South Africa presents its own unique situation that must be considered before suggesting policy or procedure for change. This would include investigating the perceptions, requirements, and objectives of the caddies themselves.

This study has some limitations, firstly due to the cross-sectional design, the relationship between musculoskeletal pain and working as a caddy should not be considered causal. The design of the study has resulted in bias by the method of data collection, including recall bias and interviewer bias. Selection bias may also have influence on the data, as a convenience sample was used. All individuals present on the day of data collection and consented to participate were included in the study, and thus were not randomly selected. This design caused information bias as information provided by those present might differ from those who were absent which could have potentially changed the findings of this study. It cannot be ruled out that the participants may not be representative of all thus making it difficult to generalize the results. Based on the information generated from this report, a larger study specific to musculoskeletal injury in caddies should be considered to further investigate the relationship and provide appropriate recommendations. In South Africa and generally across the world there have been very few studies addressing the impact of work exposures and health outcomes in caddies. 

## 5. Conclusions

Caddies are part of the expanding informal economy in South Africa. This vulnerable group of persons has been shown to have a significantly increased occurrence of musculoskeletal pain while adjusting for potentially confounding factors. As the golf industry expands so should the policy regarding the unique relationship between caddies and the golf course. It is clear that caddies represent a marginalized and vulnerable population that has a considerable increase in risk for musculoskeletal pain compared to formally employed golf course employees. Caddies should be shown methods of carrying bags to reduce additional stress on the body. In addition, golfers should be encouraged to use lighter bags, and golf courses could provide bag trolleys. Caution must be taken to ensure that new policy should not encourage golf courses to remove caddies completely as this has become their main means of income. In addition, one needs to consider and respect the direction of change considered acceptable by both golf course and caddy. There is a need for a collaboration to ensure safety and continued partnership for both. 

## Figures and Tables

**Table 1:** Socio-demographic and past history of disease distribution according to caddying and non-caddying group of male participants.

Demographics	N (%) Mean ± SD (Range)	
	Caddies (N = 249)	Non-Caddies (N = 74)
	N (%)	N (%)
Age	48.42 ± 9.23 (22 to 67)	39.68 ± 10.96 (22 to 61)
Education	N = 244	N = 71
No School	4 (1.64)	1 (1.49)
Primary School	55 (22.54)	9 (12.68)
Secondary School	165 (67.62)	55 (77.46)
Tertiary School	20 (8.19)	6 (8.45)
Primary Income	N = 249	N = 73
Yes	188 (75.50)	53 (72.60)
No	61 (24.50)	20 (27.40)
Number of Dependents	4.04 ± 2.54 (0 to 23)	4.20 ± 2.44 (0 to 14)
Monthly Income (RAND)	2849.43 ± 2506.88 (100–20,000)	5729.17 ± 5312.5 (100–40,000)
Health Indicators		
Chronic Illness	N = 249	N = 74
Yes	108 (43.37)	29 (39.19)
No	141 (56.63)	45 (60.81)
Height	63.70 ± 12.31 (41.8–121.6)	67.41 ± 11.89 (42.0–91.9)
Weight	168.77 =/ −7.97 (145.0–191.0)	167.90 =/ −8.59 (152.0–187.0)
BMI	22.39 ± 4.09 (14.8–39.7)	23.84 ± 3.40 (17.0–31.9)
Job-Related Factors		
Hours Worked Daily	5.05 ± 1.56 (4 to 12)	8.69 ± 1.62 (4 to 15)
Days Worked a Week	3.82 ± 1.38 (1–7)	5.5 ± 1.04 (2–7)

**Table 2:** Logistic Model Regression Analysis Estimating the Odds for Musculoskeletal Pain in Caddies Compared to Non-Caddies Adjusting for Covariates.

	Caddy Coefficient	Standard Error	*p*-Value	Odds Ratio (95% CI)	Sample Size
Neck Pain	1.19	4.92	0.015	3.29 (1.28, 8.89)	291
Arm Pain	1.0	0.49	0.027	2.95 (1.15, 7.86)	292
Leg Pain	1.04	0.4	0.019	2.83 (1.20, 6.89)	294
Back Pain	0.87	0.43	0.045	2.39 (1.03, 5.70)	293

Results adjusted for age, education, income, number of dependents, past history of illness, BMI and job-related factors.
